# Longitudinal associations between iron status and patient-reported outcomes in incident dialysis patients: a DOMESTICO substudy

**DOI:** 10.1007/s40620-025-02363-w

**Published:** 2025-10-02

**Authors:** Osman Mahic, Thomas S. van Lieshout, Alferso C. Abrahams, Esmee Driehuis, Ellen K. Hoogeveen, Michele F. Eisenga, Robin W. M. Vernooij, Brigit C. van Jaarsveld

**Affiliations:** 1 Department of Nephrology and Hypertension, University Medical Center Utrecht, Utrecht, The Netherlands; 2 Julius Center for Health Sciences and Primary Care, University Medical Center Utrecht, Utrecht, The Netherlands; 3 Department of Nephrology, Amsterdam UMC Location Vrije Universiteit Amsterdam, Research Institute Amsterdam Cardiovascular Sciences, Amsterdam, The Netherlands; 4 Department of Internal Medicine, Northwest Clinics, Alkmaar, The Netherlands; 5 Department of Clinical Epidemiology, Leiden University Medical Center, Leiden, The Netherlands; 6 Department of Nephrology, Leiden University Medical Center, Leiden, The Netherlands; 7 Department of Nephrology, Jeroen Bosch Hospital, ‘S-Hertogenbosch, The Netherlands; 8 Department of Nephrology, University Medical Center Groningen, Groningen, The Netherlands; 9 Nephrocare Diapriva Dialysis Center, Amsterdam, The Netherlands

**Keywords:** Kidney failure, Hemodialysis, Peritoneal dialysis, Patient-reported outcomes, Iron deficiency, Anemia

## Abstract

**Background:**

More liberal use of iron therapy is favored in dialysis patients based on lower erythropoietin need and clinical outcomes. However, it remains unclear whether higher iron stores are associated with better patient-reported outcomes. We assessed the longitudinal associations of ferritin and transferrin saturation (TSAT) levels with patient-reported outcomes in incident dialysis patients.

**Methods:**

This prospective cohort study included incident dialysis patients who had completed at least one patient-reported outcome questionnaire and had undergone a laboratory assessment (e.g., ferritin, transferrin saturation, hemoglobin) within the first year of dialysis. The primary outcome was health-related quality of life (HRQoL), measured using the 12-Item Short Form (SF-12) survey. Secondary outcomes were the presence of anemia-related symptoms, measured using the Dialysis Symptom Index. We used sequential conditional mean models to adjust for baseline and time-varying confounding.

**Results:**

We included 1069 incident dialysis patients, of whom 76% initiated hemodialysis. The mean (SD) age was 64.0 (14.2) years and 34% were female. Over a 1-year follow-up, patients with ferritin levels < 200, > 500 − 700, and > 700 ng/mL did not have a significantly different HRQoL compared to those with levels between 200 − 500 ng/mL, chosen as reference. Similarly, patients with TSAT levels < 20 or ≥ 40% did not have a significantly different HRQoL compared to those with levels between 20 − 39%. No significant differences were found in the odds of experiencing fatigue, shortness of breath, muscle cramps or restless legs between the ferritin and TSAT groups.

**Conclusion:**

Differences in iron status parameters were not associated with differences in patient-reported outcomes during the first year of dialysis. Our findings therefore suggest that decisions on iron therapy should be guided by target hemoglobin levels and clinical outcomes in dialysis patients.

**Graphical abstract:**

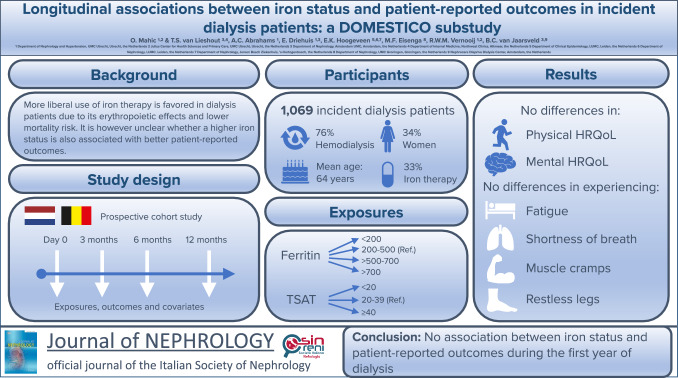

**Supplementary Information:**

The online version contains supplementary material available at 10.1007/s40620-025-02363-w.

## Introduction

Anemia is a very common complication of chronic kidney disease (CKD), affecting over half of patients with kidney failure [1]. Iron deficiency is one of the causes of anemia in CKD alongside decreased production of erythropoietin (EPO) [2]. Dialysis patients are particularly vulnerable to iron deficiency. The Kidney Disease Improving Global Outcomes (KDIGO) guidelines recommend initiating iron therapy only for anemia-related indications, i.e., increasing hemoglobin (Hb) levels and minimizing the use of EPO-stimulating agents [3]. While iron plays a key role in erythropoiesis, it also has a myriad of other physiological functions including cellular respiration and metabolism and DNA synthesis and repair [4, 5]. Low iron levels, regardless of anemia, are associated with increased risks of all-cause mortality and cardiovascular morbidity in CKD patients [6]. On the other hand, iron overload raises concerns as it can lead to oxidative stress and tissue damage [4, 5]. As a result, the KDIGO guideline cautioned against the use of iron therapy in patients with ferritin levels above 500 ng/mL or transferrin saturation (TSAT) above 30% [3]. The Proactive IV irOn Therapy in hemodiALysis patients (PIVOTAL) trial showed that the proactive administration of high-dose iron lowered the risk of mortality in hemodialysis (HD) patients compared to low-dose iron given in a reactive fashion. It is to be noted that iron therapy was withheld in patients with ferritin > 700 ng/mL and TSAT ≥ 40% due to safety concerns like vascular calcification and atherothrombosis [7]. Observational studies in HD patients indicate that even more intensive strategies than in PIVOTAL are associated with increased mortality, suggesting the need for caution to prevent dialysis patients from having excessively high iron levels [8].

In an era in which nephrology is becoming more patient-centered, patient-reported outcomes may be prioritized over clinical outcomes like mortality and morbidity [9]. While evidence from the PIVOTAL trial supports more liberal use of iron based on clinical outcomes, it is unclear whether iron levels are also associated with better patient-reported outcomes. In fact, more conservative strategies might be favored if higher iron levels negatively impact patient-reported outcomes, exemplifying that patient-reported outcomes like health-related quality of life (HRQoL) and symptom burden can help guide the treatment of anemia. A recent observational study in non-dialysis CKD patients showed that ferritin ≥ 300 ng/mL and TSAT ≤ 15% were associated with a worse physical HRQoL, while no significant differences between ferritin and TSAT levels were found in mental HRQoL [10]. However, it remains unclear what the impact of ferritin and TSAT levels on patient-reported outcomes is when considering higher cutoff levels. Moreover, there are no studies that have investigated longitudinal associations, which is relevant because iron levels can vary considerably over time. Therefore, the aim of this study was to investigate the association between iron status and patient-reported outcomes during the first year after dialysis initiation.

## Methods

### Study design

We used data from the Dutch nOcturnal and hoME dialysis Study To Improve Clinical Outcomes (DOMESTICO). DOMESTICO is a multi-center, prospective, observational cohort study in incident dialysis patients, aiming to compare home dialysis to in-center HD [11]. In this sub-study, patients had an assessment at dialysis initiation, followed by follow-up assessments at 3, 6, and 12 months. During each assessment, ferritin, TSAT, and covariates (e.g., EPO-stimulating agent and iron use) were measured, and patients were asked to complete patient-reported outcome questionnaires. The study was approved by the medical ethics committees of all participating dialysis centers, and was conducted in accordance with the Declaration of Helsinki and ICH-GCP. Reporting of the study conforms to the Strengthening the Reporting of Observational Studies in Epidemiology (STROBE) guidelines (supplementary appendix).

### Study population

Patients were enrolled in DOMESTICO if they met the following criteria: (i) ≥ 18 years old, (ii) a diagnosis of kidney failure, (iii) initiation of peritoneal dialysis (PD) or home or in-center HD between December 2017 and December 2022, (iv) no anticipated kidney transplantation within the next 3 months, and (v) a life expectancy exceeding 3 months. For this sub-study, patients were also required to have completed at least one patient-reported outcome questionnaire and to have had a laboratory measurement (e.g., ferritin, TSAT, and Hb) within the first year of dialysis to be included.

### Exposures and covariates

We used serum ferritin and TSAT to assess iron status during every assessment. We categorized patients into 4 groups depending on their ferritin levels, i.e., < 200 ng/mL, 200–500 ng/mL (reference), > 500–700 ng/mL, and > 700 ng/mL. As for TSAT, we categorized patients into 3 groups: < 20%, 20–39% (reference), and ≥ 40%. We based the lower cutoffs of 200 ng/mL for ferritin and 20% for TSAT on the general diagnostic cutoff values for iron deficiency in dialysis patients, and the cutoff of 500 ng/mL on the safety cutoff above which iron therapy is not recommended by the KDIGO guideline [3]. The upper cutoffs of 700 ng/mL and 40% were based on the safety cutoffs from the PIVOTAL trial [7]. Adjustments were made for the following baseline confounders: age, sex, estimated glomerular filtration rate (eGFR) [12] and comorbidities (Charlson Comorbidity Index: low (2 points), intermediate (3–4 points) and severe comorbidity (≥ 5 points) [13]). In addition, we adjusted for the following time-varying confounders: Hb levels, C-reactive protein (CRP) levels, and dialysis modality (PD or HD).

### Outcomes

The primary outcomes were physical and mental HRQoL, which we measured using the 12-Item Short Form (SF-12) Survey. The SF-12 captures physical and mental HRQoL through two summary scores, the physical component summary score and the mental component summary score, each ranging from 0 to 100 [14]. Secondary outcomes were the presence of pre-defined anemia-related symptoms, i.e., fatigue, shortness of breath, muscle cramps and restless legs. We measured the presence of the symptoms using the Dialysis Symptom Index questionnaire, which evaluates both the presence and severity of 30 dialysis-related symptoms. The Dialysis Symptom Index captures the presence of each symptom (yes/no) and, if present, its severity on a 5-point Likert scale [15].

### Statistical analyses

In the primary analysis, we estimated the associations of ferritin and TSAT levels with physical and mental HRQoL during the first year of dialysis. We calculated mean differences in HRQoL with 95% confidence intervals (CIs) using sequential conditional mean models. Briefly, a sequential conditional mean model estimates the short-term effect of a time-varying exposure at a given time conditional on the history of the exposure, outcome, and covariates up to that time [16]. A sequential conditional mean model can thus be used to adjust for time-varying confounding, even in case of treatment-confounder feedback. In our study, ferritin and TSAT levels changed over time, which can cause changes in HRQoL due to confounders that also change over time (e.g., CRP levels)*.* A sequential conditional mean model is a doubly robust estimator when a time-varying propensity score is included as a covariate in the outcome model [16]. We used multinomial logistic regression models to compute generalized propensity scores, which represent the conditional probabilities of being in each particular ferritin and TSAT group at a given time [17, 18]. See supplementary appendix for technical details.

For the secondary outcomes, we calculated odds ratios with 95% CIs to estimate the associations of ferritin and TSAT levels with the presence of fatigue, shortness of breath, muscle cramps, and restless legs using similar sequential conditional mean models with time-varying propensity scores. The parameters of all models were estimated using generalized estimating equations with independence working correlation matrices [16].

In our final model, we only adjusted for prior Hb levels as concurrent Hb is a potential mediator (see supplementary appendix). In an exploratory analysis, we adjusted for the history of Hb including concurrent Hb. We also performed a crude analysis in which we did not adjust for covariates but only for the history of the exposure and outcome.

We used multi-level multiple imputation based on predictive mean matching to impute missing data on ferritin, TSAT, HRQoL, symptoms and covariates under the missing at random assumption [19, 20]. See tables S1 and S2 for the number of patients with complete data at each assessment. We used the same variables as in the analysis model, as well as all auxiliary variables to generate 10 imputed datasets [21]. The propensity scores were computed and used to obtain an estimate in each dataset following imputation, which is also known as the *within* approach [22]. The results were pooled using Rubin’s rules [19, 20]. We assumed that drop-out was ignorable given the covariates in the sequential conditional mean model and did not impute data after loss to follow-up. In a sensitivity analysis, we excluded all patients who were lost to follow-up. All statistical analyses were done using R version 4.1.3 [23], with the R packages listed in the supplementary appendix.

## Results

### Baseline characteristics

We included a total of 1069 patients in this study. The mean age (SD) was 64.0 (14.2) years, with 66% being male, and the majority (76%) starting kidney replacement therapy on HD. Twenty-nine percent of patients had a severe comorbidity score, while 31% had a low comorbidity score. Regarding iron prescriptions at baseline, 11% of patients received oral iron, while 22% received intravenous (IV) iron, with ferric carboxymaltose being the most commonly used IV iron (Table 1). Baseline characteristics were similar between included and excluded patients, except for lower mental component summary scores in excluded patients (Table S3).

**Table 1:** Baseline characteristics of the total cohort at dialysis initiation

	Total patients (*n* = 1069)
Demographic	
Age, mean (SD) – yr	64.0 (14.2)
Male sex, no. (%)	705 (66)
Clinical	
Hemodialysis, no. (%)	803 (76)
Primary kidney disease, no. (%)	
Diabetic kidney disease	189 (18)
Hypertension	176 (16)
Renal vascular disease	89 (8)
Glomerulonephritis	128 (12)
Pyelonephritis	56 (5)
Polycystic kidney disease	59 (6)
Miscellaneous	194 (18)
Unknown	178 (17)
Cardiovascular disease, no. (%)	
Coronary artery disease	256 (24)
Peripheral artery disease	245 (23)
Heart failure	106 (10)
Diabetes mellitus, no. (%)	359 (34)
Malignancy, no. (%)	158 (15)
Lung disease, no. (%)	89 (8)
Charlson Comorbidity Index, no. (%)	
Low comorbidity score (2 points)	328 (31)
Intermediate comorbidity score (3–4 points)	427 (40)
Severe comorbidity score (≥ 5 points)	308 (29)
Residual diuresis (> 100 mL/day)	785 (73)
Laboratory values	
eGFR, median (IQR) – mL/min/1.73m^2^	6.4 (9.1)
Hemoglobin, mean (SD) – g/dL	10.0 (1.6)
Ferritin, median (IQR) – ng/mL	206 (299)
Transferrin saturation, median (IQR) – %	19 (14)
C-reactive protein, median (IQR) – mg/L	7.9 (23.1)
Antianemia drug use at baseline	
IV Iron, no. (%)	229 (22)
Ferric carboxymaltose, no. (%)	104 (10)
Iron sucrose, no. (%)	62 (6)
Iron isomaltoside, no. (%)	61 (6)
Oral Iron, no. (%)	116 (11)
ESA, no. (%)	592 (56)
Patient-reported outcomes	
HRQoL	
PCS, median (IQR) – 0 to 100	35.0 (13.7)
MCS, median (IQR) – 0 to 100	48.0 (16.0)
Anemia-related symptoms	
Fatigue, no. (%)	658 (82)
Shortness of breath, no. (%)	264 (33)
Muscle cramps, no. (%)	468 (59)
Restless legs, no. (%)	372 (47)

*eGFR* estimated glomerular filtration rate (2021 Chronic Kidney Disease Epidemiology Collaboration (CKD-EPI)), *IV* intravenous, *ESA* erythropoietin stimulating agent, *HRQoL* health-related quality of life, *PCS* Physical Component Summary, *MCS* Mental Component Summary. Missing values: dialysis modality, *n* = 14 (1.3%); coronary artery disease, *n* = 4 (0.4%); peripheral artery disease, *n* = 3 (0.3%); heart failure, *n* = 5 (0.5%); diabetes mellitus, *n* = 5 (0.5%); malignancy, *n* = 3 (0.3%); lung disease, *n* = 3 (0.3%); Charlson Comorbidity Index, *n* = 6 (0.6%); eGFR, *n* = 18 (1.7%); hemoglobin, *n* = 15 (1.4%); ferritin, *n* = 156 (14.6%); transferrin saturation, *n* = 247 (23.1%); iron use, *n* = 4 (0.4%); ESA use, *n* = 4 (0.4%); PCS, *n* = 351 (32.8%); MCS, *n* = 351 (32.8%); fatigue, *n* = 270 (25.3%); shortness of breath, *n* = 269 (25.2%); muscle cramps, *n* = 269 (25.2%); restless legs, *n* = 270 (25.3%)

Iron status varied among patients, with ferritin levels being distributed as follows at baseline (before imputation): 49% had levels < 200, 33% between 200–500, 7% between 500–700 and 11% > 700 ng/mL. As for TSAT, 53% had levels < 20, 40% between 20–39, and 7% ≥ 40%. Patients in the higher categories were prescribed iron therapy less frequently at baseline (Table S4 and S5).

### Iron status and HRQoL

**Fig. 1:**
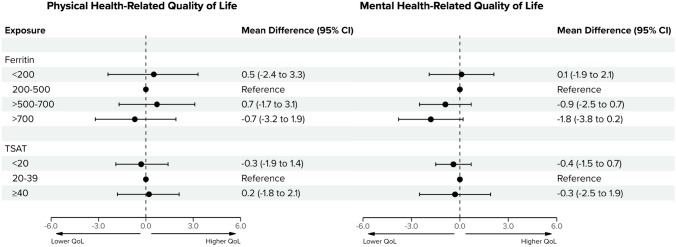
Associations of ferritin and transferrin saturation with health-related quality of life during the first year of dialysis. Mean differences represent the longitudinal associations of different ferritin and transferrin saturation (TSAT) levels with physical and mental health-related quality of life during the first year of dialysis. Physical and mental health-related quality of life refer to the Physical Component Summary (PCS) and Mental Component Summary (MCS) scores from the 12-Item Short Form (SF-12) survey. Adjustments were made for the following baseline confounders: age, sex, estimated glomerular filtration rate, Charlson Comorbidity Index, and the following time-varying confounders: hemoglobin, C-reactive protein, and dialysis modality

The response rates for the SF-12 Survey at baseline, 3 months, 6 months, and 12 months were 75%, 69%, 71%, and 72%, respectively (Table S1). Mental component summary scores were notably higher than physical component summary scores at baseline, with median (IQR) values of 48.0 (16.0) and 35.0 (13.7), respectively. Patients with ferritin levels < 200, > 500 − 700, and > 700 ng/mL did not have significantly different physical component summary or mental component summary scores compared to those with levels between 200 − 500 ng/mL, which was our reference category (Fig. 1). Patients with ferritin levels > 700 ng/mL had lower physical component summary and mental component summary scores, but the differences were not statistically significant. The mean differences were −0.7 (95% CI −3.2 to 1.9), and −1.8 (95% CI −3.8 to 0.2), respectively (Fig. 1). All effect sizes were similar when adjusted for concurrent Hb levels or when no covariates were adjusted for (Tables S6 and S7).

Regarding TSAT, patients with levels < 20 and ≥ 40% also did not have significantly different physical component summary or mental component summary scores to those with levels between 20–39%. For physical component summary scores, the mean differences were −0.3 (95% CI −1.9 to 1.4) and 0.2 (95% CI −1.8 to 2.1), respectively. For mental component summary scores, the mean differences were −0.4 (95% CI −1.5 to 0.7) and −0.3 (95% CI −2.5 to 1.9), respectively (Fig. 1). As with ferritin levels, all effect sizes were similar in both the exploratory analysis and crude analysis (Tables S6 and S7).

### Iron status and anemia-related symptoms

**Fig. 2:**
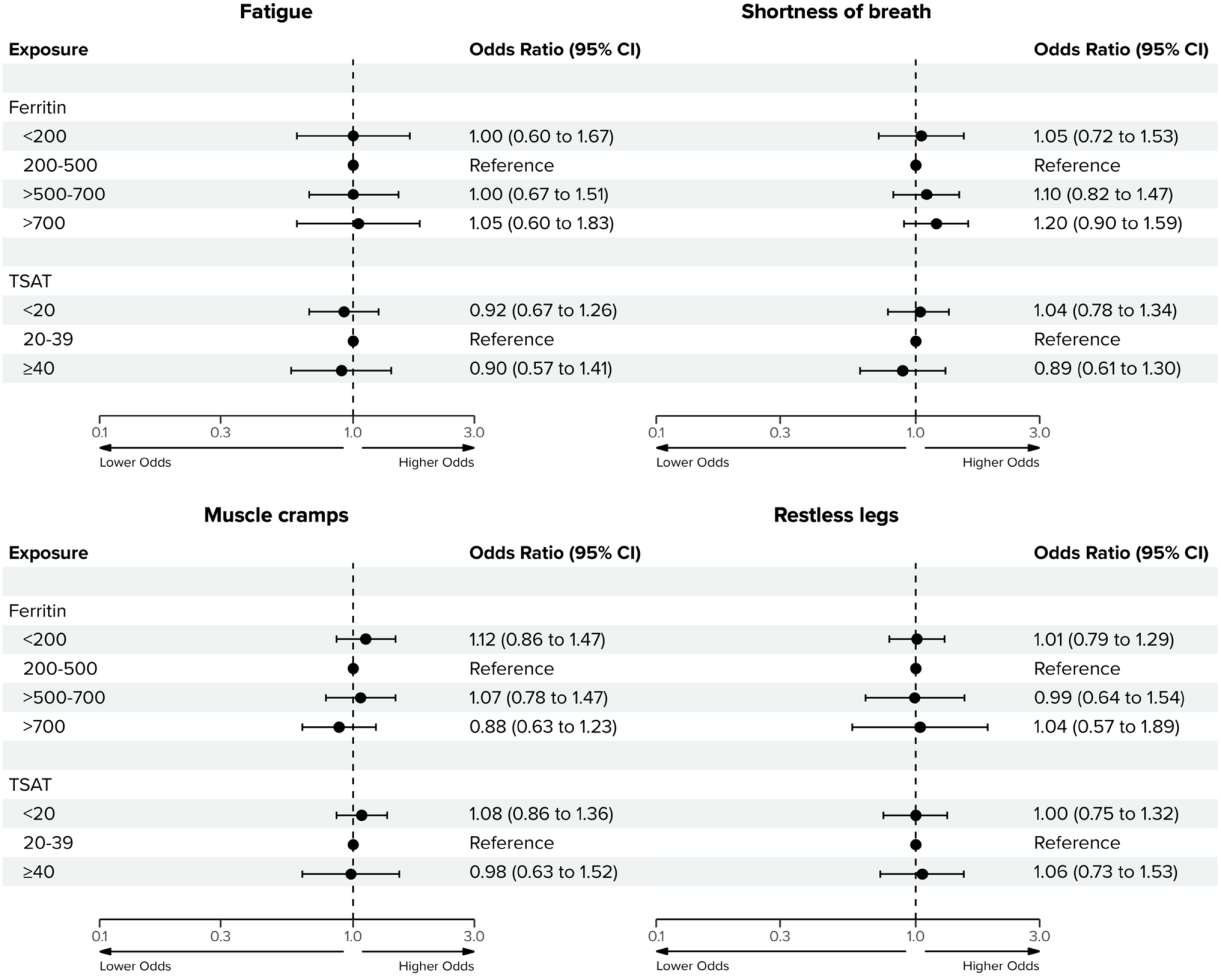
Associations of ferritin and transferrin saturation with the presence of anemia-related symptoms during the first year of dialysis. Odds ratios represent the longitudinal associations of different ferritin and transferrin saturation (TSAT) levels with the presence of fatigue, shortness of breath, muscle cramps and restless legs during the first year of dialysis. Adjustments were made for the following baseline confounders: age, sex, estimated glomerular filtration rate, Charlson Comorbidity Index, and the following time-varying confounders: hemoglobin, C-reactive protein, and dialysis modality

The response rates for the Dialysis Symptom Index questionnaire at baseline, 3 months, 6 months, and 12 months were 75%, 69%, 71%, and 71%, respectively (Table S1). At baseline, fatigue was the most commonly reported symptom (82%), followed by muscle cramps (59%), restless legs (47%), and shortness of breath (33%). We found no statistically significant differences in the odds of having fatigue, shortness of breath, muscle cramps or restless legs between the ferritin and TSAT groups. Figure 2 presents the odds ratios across all ferritin and TSAT groups. All results were similar when adjusted for Hb (Table S6). Likewise, the crude analysis yielded similar estimates (Table S7).

### Sensitivity analysis

At the end of the 12-month study period, 267 (25%) patients were lost to follow-up. Of those lost to follow-up, the majority underwent kidney transplantation (36%) or died (25%). Baseline characteristics were comparable in those patients who were lost to follow-up versus those who were not (Table S8). Our results were also consistent in the sensitivity analysis, in which we excluded all of the patients who were lost to follow-up (Table S9).

## Discussion

In our nationwide observational cohort study, we found no association of iron status with HRQoL, nor with the presence of fatigue, shortness of breath, muscle cramps or restless leg syndrome during the first year of dialysis. Importantly, our results were similar after adjusting for key baseline and time-varying confounders, such as comorbidities and CRP levels, compared to when no additional adjustments for confounders were made.

At the start of dialysis, the majority of patients had a low iron status; the median ferritin and TSAT levels were 206 ng/mL and 19%, respectively. However, only 33% of patients were using any form of iron therapy at baseline. There is considerable variation in iron use during dialysis across countries, leading to differences in ferritin and TSAT levels. For example, in the USA, over 40% of prevalent HD patients had ferritin levels above 800 ng/mL, which is much higher than in our dialysis cohort [24, 25]. While more liberal dosing strategies are now considered superior following the PIVOTAL trial, a degree of caution is warranted in patients with excessively high iron status. Our cutoffs for ferritin and TSAT were based on current leading evidence from the PIVOTAL trial and the KDIGO guideline and reflect the latest prescribing practices. Although the lower cutoffs to define iron deficiency have been scrutinized, these definitions generally rely on thresholds of 200 ng/mL for ferritin and 20% for TSAT in HD patients [3, 26], which are the cutoffs we used.

Maintaining adequate iron levels is crucial to prevent anemia, increased EPO-stimulating agent dose requirements, and blood transfusions. However, it is also important to consider HRQoL when managing iron levels. Since iron plays a crucial role in muscle, brain, and cardiovascular health, it could be speculated that iron status would be an important factor determining HRQoL in the current patient setting [27, 28]. Surprisingly, in our study, higher ferritin and TSAT levels were not associated with better HRQoL, and levels exceeding safety thresholds had no impact on HRQoL either. Importantly, our results remained consistent after adjusting for Hb levels, suggesting that iron, either directly or through Hb, does not impact HRQoL in dialysis patients. An observational study in non-dialysis CKD patients from France, USA and Brazil (CKDopps) showed that ferritin levels ≥ 300 ng/mL, and TSAT levels ≤ 15% were associated with a worse physical HRQoL [10]. It is well conceivable that the effect of iron differs between non-dialysis and dialysis CKD patients, as the latter experience more severe health impairments and symptoms [29]. However, there are a few other important differences between our study and the study in non-dialysis CKD patients. For instance, the cutoffs used in the CKDopps study were based on the heart failure population and are therefore more conservative than ours, which could result in different findings. Moreover, another possible difference that could help explain our different findings is that ferritin and TSAT levels were treated as time-fixed in the non-dialysis CKD study, despite iron levels varying considerably over time, which could introduce bias [30]. Ultimately, very modest associations were found in HRQoL (< 2 points) in this study that may also be attributed to the larger sample size of 2513 [10].

While HRQoL is widely recognized as a key measure of patient well-being, it is comprised of various factors, including symptoms that may affect overall quality of life. To our knowledge, no prior research has investigated the association between iron status and specific symptoms in either dialysis or non-dialysis CKD patients. In our study, we also found no association between iron status and the commonly reported anemia-related symptoms like fatigue, shortness of breath, muscle cramps and restless legs. Although iron plays important physiological roles [4, 5], its effects do not appear to translate into improvements of symptoms in dialysis patients.

Our study has several strengths. We used longitudinal data on ferritin and TSAT, which allowed us to investigate longitudinal associations in a large cohort of HD and PD patients. We used robust statistical approaches to adjust for both baseline and time-varying confounding. Moreover, we based the cutoffs for ferritin and TSAT on the latest evidence, and thus chose those that are most useful for current clinical practice. However, our study also has a few limitations. Excluded patients had lower mental component summary scores, likely due to more missing data, but given that selection was not related to the exposures, a selection bias appears unlikely. Despite adjusting for key confounders, we cannot rule out residual confounding due to the observational nature of our study, as not all potential confounders, such as nutritional status, were measured. Due to the variability of missing data across time points and the nature of our analytic approach, we were unable to conduct a complete case analysis using non-imputed data. We were also unable to achieve convergence with item-level imputation for HRQoL and had to impute at the outcome level, which may introduce some bias. Similarly, despite the use of strong auxiliary variables for imputation, we cannot rule out that some data may be missing not at random, especially in the case of patient-reported outcomes. However, the number of missing responses per item varied by no more than 5% (Table S1), suggesting that the risk of bias may be limited. Although serum ferritin and TSAT are the most widely used markers for assessing iron status in clinical practice, we recognize that accurately determining iron stores remains challenging and that more invasive diagnostic tests (e.g., bone marrow biopsy) may be more reliable but not clinically feasible. Lastly, since we only focused on the first year of dialysis, we were not able to capture potential effects of iron on patient-reported outcomes beyond this period.

Future research could therefore explore longer follow-up periods, such as two years. Given that we observed a trend toward lower mental component summary scores with increasing ferritin levels, future studies could explore the impact of using even higher cutoff thresholds for ferritin. Additionally, since our study did not compare treatment strategies, future studies could examine the effects of iron treatment strategies on patient-reported outcomes, ideally through a randomized trial or an emulation thereof. Lastly, future studies should preferably include a greater number of older patients, those with limited life expectancy and those not waitlisted for transplantation, as these subgroups may experience greater symptom burden and reduced quality of life.

To conclude, we found no association of iron status with patient-reported outcomes in more than 1000 patients during the first year after dialysis initiation. Our findings suggest that there may be no benefits of having a higher iron status for patient-reported outcomes, and that decisions on iron therapy in dialysis patients should therefore be guided by clinical and biochemical outcomes like Hb levels and EPO-stimulating agent requirements.

## Supplementary Information

Below is the link to the electronic supplementary material.

Supplementary file1 (DOCX 141 KB)

## Data Availability

The data that support the findings of this study are available from the corresponding author upon reasonable request and with permission of the DOMESTICO steering committee.
