# Supplementary material for: Longitudinal associations between iron status and patient-reported outcomes in incident dialysis patients: a DOMESTICO substudy
Source: J Nephrol. 2025 Oct 2;38(9):2641–50. doi: 10.1007/s40620-025-02363-w (PMC12712045; doi:10.1007/s40620-025-02363-w)
Supplement: Supplementary file 1 — Supplementary file1 (DOCX 141 KB) [file 40620_2025_2363_MOESM1_ESM.docx]

**SUPPLEMENTARY FILES**

**Longitudinal associations between iron status and patient-reported outcomes in incident dialysis patients: a DOMESTICO study**

O. Mahic & T.S. van Lieshout, A.C. Abrahams, E. Driehuis, E.K. Hoogeveen, M.F. Eisenga, R.W.M. Vernooij, B.C. van Jaarsveld

**Supplementary material.** STROBE checklist for cohort studies

**Supplementary methods.** Technical details on statistical analysis

**Supplementary material.** Software and R packages

**Table S1.** Number of patients with complete data on outcomes

**Table S2.** Number of patients with complete data on exposures and covariates

**Table S3.** Baseline characteristics: included vs. excluded patients

**Table S4.** Baseline characteristics of incident dialysis patients stratified by ferritin levels

**Table S5.** Baseline characteristics of incident dialysis patients stratified by TSAT levels

**Table S6.** Exploratory analysis of the association of iron status with HRQoL and anemia-related symptoms with additional adjustment for concurrent hemoglobin

**Table S7.** Crude association of iron status with HRQoL and anemia-related symptoms without adjustment for covariates

**Table S8.** Baseline characteristics: lost to follow-up vs. not lost to follow-up

**Table S9.** Sensitivity analysis of the association of iron status with HRQoL and anemia-related symptoms in patients with complete follow-up

**Supplementary material.** STROBE checklist for cohort studies

STROBE Statement—Checklist of items that should be included in reports of ***cohort studies***

|  | Item No | Recommendation | Page No |
| --- | --- | --- | --- |
| **Title and abstract** | 1 | (*a*) Indicate the study’s design with a commonly used term in the title or the abstract | 2 |
|  |  | (*b*) Provide in the abstract an informative and balanced summary of what was done and what was found |  |
| Introduction | | | |
| Background/rationale | 2 | Explain the scientific background and rationale for the investigation being reported | 3 |
| Objectives | 3 | State specific objectives, including any prespecified hypotheses | 3 |
| Methods | | | |
| Study design | 4 | Present key elements of study design early in the paper | 4 |
| Setting | 5 | Describe the setting, locations, and relevant dates, including periods of recruitment, exposure, follow-up, and data collection | 4 |
| Participants | 6 | (*a*) Give the eligibility criteria, and the sources and methods of selection of participants. Describe methods of follow-up | 4 |
|  |  | (*b*) For matched studies, give matching criteria and number of exposed and unexposed |  |
| Variables | 7 | Clearly define all outcomes, exposures, predictors, potential confounders, and effect modifiers. Give diagnostic criteria, if applicable | 4,5 |
| Data sources/ measurement | 8* | For each variable of interest, give sources of data and details of methods of assessment (measurement). Describe comparability of assessment methods if there is more than one group | 4,5 |
| Bias | 9 | Describe any efforts to address potential sources of bias | 5,6 |
| Study size | 10 | Explain how the study size was arrived at | 4 |
| Quantitative variables | 11 | Explain how quantitative variables were handled in the analyses. If applicable, describe which groupings were chosen and why | 4,5 |
| Statistical methods | 12 | (*a*) Describe all statistical methods, including those used to control for confounding | 5,6 |
|  |  | (*b*) Describe any methods used to examine subgroups and interactions |  |
|  |  | (*c*) Explain how missing data were addressed |  |
|  |  | (*d*) If applicable, explain how loss to follow-up was addressed |  |
|  |  | (*e*) Describe any sensitivity analyses |  |
| Results | | |  |
| Participants | 13* | (a) Report numbers of individuals at each stage of study—eg numbers potentially eligible, examined for eligibility, confirmed eligible, included in the study, completing follow-up, and analysed | 6,10 |
|  |  | (b) Give reasons for non-participation at each stage |  |
|  |  | (c) Consider use of a flow diagram |  |
| Descriptive data | 14* | (a) Give characteristics of study participants (eg demographic, clinical, social) and information on exposures and potential confounders | 6 |
|  |  | (b) Indicate number of participants with missing data for each variable of interest |  |
|  |  | (c) Summarise follow-up time (eg, average and total amount) |  |
| Outcome data | 15* | Report numbers of outcome events or summary measures over time | 8,9 |

| Main results | 16 | (*a*) Give unadjusted estimates and, if applicable, confounder-adjusted estimates and their precision (eg, 95% confidence interval). Make clear which confounders were adjusted for and why they were included | 8,9 |
| --- | --- | --- | --- |
|  |  | (*b*) Report category boundaries when continuous variables were categorized |  |
|  |  | (*c*) If relevant, consider translating estimates of relative risk into absolute risk for a meaningful time period |  |
| Other analyses | 17 | Report other analyses done—eg analyses of subgroups and interactions, and sensitivity analyses | 10 |
| Discussion | | | |
| Key results | 18 | Summarise key results with reference to study objectives | 11 |
| Limitations | 19 | Discuss limitations of the study, taking into account sources of potential bias or imprecision. Discuss both direction and magnitude of any potential bias | 12 |
| Interpretation | 20 | Give a cautious overall interpretation of results considering objectives, limitations, multiplicity of analyses, results from similar studies, and other relevant evidence | 12 |
| Generalisability | 21 | Discuss the generalisability (external validity) of the study results | 12 |
| Other information | | | |
| Funding | 22 | Give the source of funding and the role of the funders for the present study and, if applicable, for the original study on which the present article is based | 14 |

*Give information separately for exposed and unexposed groups.

**Note:** An Explanation and Elaboration article discusses each checklist item and gives methodological background and published examples of transparent reporting. The STROBE checklist is best used in conjunction with this article (freely available on the Web sites of PLoS Medicine at http://www.plosmedicine.org/, Annals of Internal Medicine at http://www.annals.org/, and Epidemiology at http://www.epidem.com/). Information on the STROBE Initiative is available at <http://www.strobe-statement.org>.

**Supplementary methods.** Technical details on statistical analysis

We used a sequential conditional mean model to estimate the following conditional expectation at a given time:

$$\mathbb{E}\left[ Y_{t} \right|\bar{X}_{t}, \bar{Y}_{t-1}, {\bar{\boldsymbol{L}}}_{t},\hat{GPS_{t}}]= \theta_{0}+\theta_{1}X_{t}+\theta_{2}X_{t-1}+\theta_{3}Y_{t-1}+\theta_{4}^{T}\boldsymbol{L}_{t}+\theta_{5}\hat{GPS_{t}}$$

Where Y represents the outcome, X the exposure and **L** a vector of covariates. The subscript t denotes time of measurement, and the overbar the history up to t. GPS represents the propensity score, or the conditional probability of exposure at t.

$\theta_{1}$captures the *short-term effect* of $X$ on $Y$. Such model is a doubly-robust estimator due to the inclusion of a (time-varying) propensity score. Given that $X$ $\in$ {1,….., $K$}, we computed the following generalized propensity scores using multinomial logistic regression:

$$GPS_{t}=\Pr\left[ X_{t}=k \right|\bar{X}_{t-1}, \bar{Y}_{t-1}, {\bar{\boldsymbol{L}}}_{t}]$$

Where the class probabilities were estimated using the softmax function:

$$\Pr\left[ X_{t}=k \right|\boldsymbol{V}]= \frac{1}{\sum_{j=1}^{K} e^{\theta_{j}^{'}\boldsymbol{V}}} e^{\theta_{\boldsymbol{k}}^{'}\boldsymbol{V}}$$

With **V** denoting the covariate vector ​($\bar{X}_{t-1}, \bar{Y}_{t-1}, {\bar{\boldsymbol{L}}}_{t}$).

Given that $\sum_{k=1}^{K} \Pr\left[ X_{t}=k \right|\boldsymbol{V}]=1$, we only included $K-1$ propensity scores in the SCMM.

**
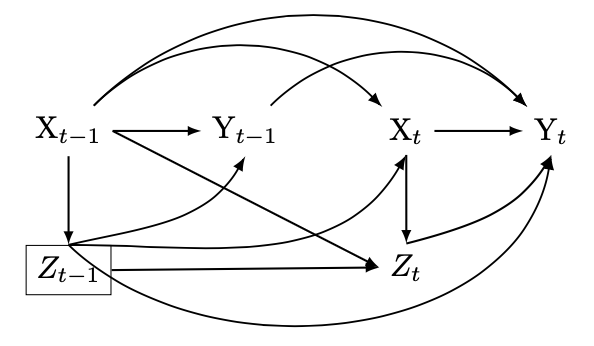
**Since hemoglobin (denoted as $Z$) is a potential mediator at time $t$, the vector of covariates in $\boldsymbol{L}$ only included its history up to, but not including time $t$. See the following directed acyclic graph (DAG) for an illustration:

In our exploratory analysis, we conditioned on both $Z_{t-1}$ and $Z_{t}$.

**Supplementary material.** Software and R packages

The following R (4.1.3) packages were used:

- dplyr (1.1.2) for data wrangling
- nephro (1.4) for eGFR calculation
- table1 (1.4.2) for baseline data summaries
- mice (3.16.8), miceadds (3.16.8), and lme4 (1.1.32) for multiple imputation
- nnet (7.3.18) for propensity score estimation
- geepack (1.3.9) for SCMM estimation
- forester (0.2.0) for visualization

**Table S1.** Number of patients with complete data on outcomes

|  |  | Baseline | 3 months | 6 months | 12 months |
| --- | --- | --- | --- | --- | --- |
|  | **Number of patients** | **1069** | **1069** | **990** | **803** |
| Short-Form 12 Survey | SF-12 1 | 799 | 742 | 703 | 580 |
|  | SF-12 2 | 802 | 733 | 705 | 576 |
|  | SF-12 3 | 773 | 716 | 677 | 550 |
|  | SF-12 4 | 796 | 729 | 694 | 573 |
|  | SF-12 5 | 790 | 726 | 692 | 568 |
|  | SF-12 6 | 790 | 733 | 690 | 565 |
|  | SF-12 7 | 781 | 714 | 686 | 559 |
|  | SF-12 8 | 791 | 733 | 697 | 566 |
|  | SF-12 9 | 790 | 733 | 700 | 566 |
|  | SF-12 10 | 788 | 732 | 698 | 563 |
|  | SF-12 11 | 792 | 736 | 698 | 568 |
|  | SF-12 12 | 794 | 735 | 697 | 563 |
|  | Total score (PCS) | 718 | 673 | 642 | 509 |
|  | Total score (MCS) | 718 | 673 | 642 | 509 |
| Dialysis Symptom Index | DSI_6 (Muscle cramps) | 800 | 742 | 704 | 574 |
|  | DSI_8 (Shortness of breath) | 800 | 741 | 702 | 574 |
|  | DSI_10 (Restless legs) | 799 | 737 | 703 | 574 |
|  | DSI_12 (Fatigue) | 799 | 741 | 701 | 572 |

^PCS = Physical Component Summary, MCS = Mental Component Summary.^

**Table S2.** Number of patients with complete data on exposures and covariates

|  | Baseline | 3 months | 6 months | 12 months |
| --- | --- | --- | --- | --- |
| **Number of patients** | **1069** | **1069** | **990** | **803** |
| Ferritin | 913 | 942 | 889 | 728 |
| Transferrin saturation | 822 | 876 | 841 | 704 |
| Hemoglobin | 1054 | 1029 | 932 | 737 |
| C-reactive protein | 748 | 597 | 548 | 433 |
| Dialysis modality | 1055 | 1058 | 985 | 799 |
| Charlson Comorbidity Index | 1063 | - | - | - |
| eGFR | 1051 | - | - | - |
| Sex | 1069 | - | - | - |
| Age | 1069 | - | - | - |

^eGFR = estimated glomerular filtration rate (2021 Chronic Kidney Disease Epidemiology Collaboration (CKD-EPI))^

**Table S3.** Baseline characteristics: included vs. excluded patients

|  | **Included patients**  (n=1069) | **Excluded patients**  (n=444) |
| --- | --- | --- |
| **Demographic** |  |  |
| Age, mean (SD) – yr. | 64 (14) | 63 (15) |
| Male sex, no. (%) | 705 (66) | 295 (66) |
| **Clinical** |  |  |
| Hemodialysis, no. (%) | 803 (76) | 311 (72) |
| *Primary kidney disease, no. (%)* |  |  |
| Diabetic kidney disease | 189 (18) | 86 (19) |
| Hypertension | 176 (16) | 79 (18) |
| Renal vascular disease | 89 (8) | 33 (7) |
| Glomerulonephritis | 128 (12) | 54 (12) |
| Pyelonephritis | 56 (5) | 19 (4) |
| Polycystic kidney disease | 59 (6) | 23 (5) |
| Miscellaneous | 194 (18) | 68 (15) |
| Unknown | 178 (17) | 82 (19) |
| *Cardiovascular disease, no. (%)* |  |  |
| Coronary artery disease | 256 (24) | 106 (25) |
| Peripheral artery disease | 245 (23) | 105 (24) |
| Heart failure | 106 (10) | 47 (11) |
| Diabetes mellitus, no. (%) | 359 (34) | 171 (40) |
| Malignancy, no. (%) | 158 (15) | 67 (16) |
| Lung disease, no. (%) | 89 (8) | 38 (9) |
| *Charlson Comorbidity Index, no. (%)* |  |  |
| Low comorbidity score (2 points) | 328 (31) | 126 (30) |
| Intermediate comorbidity score (3-4 points) | 427 (40) | 154 (36) |
| Severe comorbidity score (≥5 points) | 308 (29) | 144 (34) |
| Residual diuresis (>100 mL/day) | 785 (73) | 311 (70) |
| **Laboratory values** |  |  |
| eGFR, median (IQR) – mL/min/1.73m^2^ | 6.4 (9.1) | 5.8 (9.5) |
| Hemoglobin, mean (SD) – g/dL | 10.0 (1.6) | 10.0 (2.3) |
| Ferritin, median (IQR) – ng/mL | 206 (299) | 205 (267) |
| Transferrin saturation, median (IQR) – % | 19 (14) | 18 (14) |
| C-reactive protein, median (IQR) – mg/L | 7.9 (23.1) | 9.0 (27.9) |
| **Antianemia drugs** |  |  |
| IV Iron, no. (%) | 229 (22) | 81 (19) |
| Ferric carboxymaltose, no. (%) | 104 (10) | 38 (9) |
| Iron sucrose, no. (%) | 62 (6) | 7 (2) |
| Iron isomaltoside, no. (%) | 61 (6) | 36 (8) |
| Oral Iron, no. (%) | 116 (11) | 47 (11) |
| ESA, no. (%) | 592 (56) | 247 (57) |
| **Patient-reported outcomes** |  |  |
| *HRQoL* |  |  |
| PCS, median (IQR) – 0 to 100 | 35.0 (13.7) | 34.7 (13.6) |
| MCS, median (IQR) – 0 to 100 | 48.0 (16.0) | 45.8 (18.3) |
| *Anemia-related symptoms* |  |  |
| Fatigue, no. (%) | 658 (82) | 164 (82) |
| Shortness of breath, no. (%) | 264 (33) | 67 (34) |
| Muscle cramps, no. (%) | 468 (59) | 116 (58) |
| Restless legs, no. (%) | 372 (47) | 90 (45) |

^eGFR = estimated glomerular filtration rate (2021 Chronic Kidney Disease Epidemiology Collaboration (CKD-EPI)), IV= intravenous, ESA = erythropoietin stimulating agents, HRQoL = health-related quality of life, PCS = Physical Component Summary, MCS = Mental Component Summary.^

**Table S4.** Baseline characteristics of incident dialysis patients stratified by ferritin levels

|  | **<200**  (n=444) | **200-500**  (n=306) | **>500-700**  (n=64) | **>700**  (n=99) |
| --- | --- | --- | --- | --- |
| **Demographic** |  |  |  |  |
| Age, mean (SD) – yr. | 64 (14) | 64 (14) | 63 (13) | 63 (16) |
| Male sex, no. (%) | 276 (62) | 224 (73) | 42 (66) | 64 (65) |
| **Clinical** |  |  |  |  |
| Hemodialysis, no. (%) | 345 (79) | 224 (74) | 47 (73) | 80 (81) |
| *Primary kidney disease, no. (%)* |  |  |  |  |
| Diabetic kidney disease | 87 (20) | 63 (21) | 10 (16) | 9 (9) |
| Hypertension | 73 (16) | 43 (14) | 9 (14) | 18 (18) |
| Renal vascular disease | 35 (8) | 23 (8) | 6 (9) | 8 (8) |
| Glomerulonephritis | 57 (13) | 36 (12) | 7 (11) | 9 (9) |
| Pyelonephritis | 30 (7) | 12 (4) | 2 (3) | 5 (5) |
| Polycystic kidney disease | 29 (7) | 14 (5) | 3 (5) | 2 (2) |
| Miscellaneous | 69 (16) | 51 (17) | 16 (25) | 29 (29) |
| Unknown | 64 (14) | 64 (21) | 11 (17) | 19 (19) |
| *Cardiovascular disease, no. (%)* |  |  |  |  |
| Coronary artery disease | 107 (24) | 77 (25) | 10 (16) | 22 (22) |
| Peripheral artery disease | 104 (23) | 72 (24) | 12 (19) | 16 (16) |
| Heart failure | 42 (9) | 39 (13) | 5 (8) | 8 (8) |
| Diabetes mellitus, no. (%) | 166 (37) | 107 (35) | 17 (27) | 25 (26) |
| Malignancy, no. (%) | 59 (13) | 47 (15) | 7 (11) | 25 (26) |
| Lung disease, no. (%) | 40 (9) | 24 (8) | 5 (8) | 11 (11) |
| *Charlson Comorbidity Index, no. (%)* |  |  |  |  |
| Low comorbidity score (2 points) | 147 (33) | 80 (26) | 25 (40) | 25 (26) |
| Intermediate comorbidity score (3-4 points) | 162 (37) | 128 (42) | 23 (37) | 47 (48) |
| Severe comorbidity score (≥5 points) | 134 (30) | 96 (32) | 15 (24) | 26 (27) |
| Residual diuresis (>100 mL/day) | 333 (75) | 226 (74) | 46 (72) | 71 (72) |
| **Laboratory values** |  |  |  |  |
| eGFR, median (IQR) – mL/min/1.73m^2^ | 6.6 (9.1) | 6.4 (8.8) | 6.3 (9.7) | 4.8 (8.3) |
| Hemoglobin, mean (SD) – g/dL | 10.1 (1.5) | 9.8 (1.6) | 9.8 (1.5) | 9.2 (1.9) |
| Ferritin, median (IQR) – ng/mL | 99 (81) | 302 (144) | 582 (104) | 1040 (641) |
| Transferrin saturation, median (IQR) – % | 17 (10) | 21 (15) | 24 (14) | 23 (23) |
| C-reactive protein, median (IQR) – mg/L | 5.0 (12.0) | 8.0 (21.0) | 11.5 (40.8) | 28.5 (76.7) |
| **Antianemia drugs** |  |  |  |  |
| IV Iron, no. (%) | 117 (26) | 66 (22) | 5 (8) | 10 (10) |
| Ferric carboxymaltose, no. (%) | 58 (13) | 28 (9) | 2 (3) | 4 (4) |
| Iron sucrose, no. (%) | 30 (7) | 17 (6) | 2 (3) | 4 (4) |
| Iron isomaltoside, no. (%) | 29 (7) | 21 (7) | 1 (2) | 2 (2) |
| Oral Iron, no. (%) | 65 (15) | 30 (10) | 6 (9) | 8 (8) |
| ESA, no. (%) | 256 (58) | 168 (55) | 39 (61) | 54 (55) |
| **Patient-reported outcomes** |  |  |  |  |
| *HRQoL* |  |  |  |  |
| PCS, median (IQR) – 0 to 100 | 35.7 (14.0) | 34.8 (14.4) | 34.2 (14.6) | 33.1 (10.7) |
| MCS, median (IQR) – 0 to 100 | 48.4 (14.7) | 48.2 (14.2) | 39.5 (13.2) | 46.5 (17.3) |
| *Anemia-related symptoms* |  |  |  |  |
| Fatigue, no. (%) | 258 (79) | 192 (84) | 41 (87) | 59 (79) |
| Shortness of breath, no. (%) | 106 (32) | 75 (33) | 23 (49) | 24 (32) |
| Muscle cramps, no. (%) | 201 (61) | 135 (59) | 26 (55) | 38 (51) |
| Restless legs, no. (%) | 152 (46) | 101 (44) | 24 (51) | 32 (43) |

^eGFR = estimated glomerular filtration rate (2021 Chronic Kidney Disease Epidemiology Collaboration (CKD-EPI)), IV= intravenous, ESA = erythropoietin stimulating agents, HRQoL = health-related quality of life, PCS = Physical Component Summary, MCS = Mental Component Summary.^

Note: baseline characteristics represent values before imputation. The groups are therefore smaller in size compared to those used in the analysis after imputation.

**Table S5.** Baseline characteristics of incident dialysis patients stratified by TSAT levels

|  | **<20**  (n=435) | **20-39**  (n=329) | **≥40**  (n=58) |
| --- | --- | --- | --- |
| **Demographic** |  |  |  |
| Age, mean (SD) – yr. | 65 (14) | 64 (14) | 60 (16) |
| Male sex, no. (%) | 279 (64) | 228 (69) | 38 (66) |
| **Clinical** |  |  |  |
| Hemodialysis, no. (%) | 341 (79) | 237 (73) | 42 (74) |
| *Primary kidney disease, no. (%)* |  |  |  |
| Diabetic kidney disease | 95 (22) | 50 (15) | 5 (9) |
| Hypertension | 64 (15) | 52 (16) | 9 (16) |
| Renal vascular disease | 36 (8) | 29 (9) | 4 (7) |
| Glomerulonephritis | 55 (13) | 40 (12) | 6 (10) |
| Pyelonephritis | 22 (5) | 21 (6) | 2 (3) |
| Polycystic kidney disease | 22 (5) | 16 (5) | 2 (3) |
| Miscellaneous | 79 (18) | 55 (17) | 17 (29) |
| Unknown | 62 (14) | 66 (20) | 13 (22) |
| *Cardiovascular disease, no. (%)* |  |  |  |
| Coronary artery disease | 118 (27) | 74 (22) | 9 (16) |
| Peripheral artery disease | 110 (25) | 73 (22) | 10 (18) |
| Heart failure | 54 (12) | 31 (9) | 1 (2) |
| Diabetes mellitus, no. (%) | 161 (37) | 109 (33) | 16 (28) |
| Malignancy, no. (%) | 72 (17) | 44 (13) | 11 (19) |
| Lung disease, no. (%) | 41 (9) | 30 (9) | 1 (2) |
| *Charlson Comorbidity Index, no. (%)* |  |  |  |
| Low comorbidity score (2 points) | 118 (27) | 107 (33) | 20 (35) |
| Intermediate comorbidity score (3-4 points) | 180 (42) | 121 (37) | 23 (40) |
| Severe comorbidity score (≥5 points) | 134 (31) | 100 (30) | 14 (25) |
| Residual diuresis (>100 mL/day) | 319 (73) | 249 (76) | 41 (71) |
| **Laboratory values** |  |  |  |
| eGFR, median (IQR) – mL/min/1.73m^2^ | 6.4 (9.0) | 6.6 (9.4) | 5.9 (8.3) |
| Hemoglobin, mean (SD) – g/dL | 9.7 (1.4) | 10.3 (1.7) | 9.7 (1.8) |
| Ferritin, median (IQR) – ng/mL | 157 (240) | 232 (278) | 470 (648) |
| Transferrin saturation, median (IQR) – % | 13 (6) | 26 (8) | 47 (11) |
| C-reactive protein, median (IQR) – mg/L | 9.9 (33.8) | 4.3 (12.0) | 6.0 (18.5) |
| **Antianemia drugs** |  |  |  |
| IV Iron, no. (%) | 104 (24) | 63 (19) | 4 (7) |
| Ferric carboxymaltose, no. (%) | 56 (13) | 30 (9) | 1 (2) |
| Iron sucrose, no. (%) | 25 (6) | 23 (7) | 2 (3) |
| Iron isomaltoside, no. (%) | 23 (5) | 10 (3) | 2 (3) |
| Oral Iron, no. (%) | 53 (12) | 42 (13) | 4 (7) |
| ESA, no. (%) | 262 (61) | 162 (49) | 29 (50) |
| **Patient-reported outcomes** |  |  |  |
| *HRQoL* |  |  |  |
| PCS, median (IQR) – 0 to 100 | 34.0 (14.4) | 35.0 (13.8) | 39.4 (11.1) |
| MCS, median (IQR) – 0 to 100 | 47.7 (16.6) | 47.8 (14.1) | 47.2 (17.6) |
| *Anemia-related symptoms* |  |  |  |
| Fatigue, no. (%) | 255 (81) | 212 (82) | 31 (76) |
| Shortness of breath, no. (%) | 107 (34) | 78 (30) | 16 (39) |
| Muscle cramps, no. (%) | 190 (61) | 150 (58) | 23 (56) |
| Restless legs, no. (%) | 154 (49) | 109 (42) | 16 (39) |

^eGFR = estimated glomerular filtration rate (2021 Chronic Kidney Disease Epidemiology Collaboration (CKD-EPI)), IV= intravenous, ESA = erythropoietin stimulating agents, HRQoL = health-related quality of life, PCS = Physical Component Summary, MCS = Mental Component Summary.^

Note: baseline characteristics represent values before imputation. The groups are therefore smaller in size compared to those used in the analysis after imputation.

**Table S6.** Exploratory analysis of the association of iron status with HRQoL and anemia-related symptoms with additional adjustment for concurrent hemoglobin

|  | **Physical HRQoL** | | | **Mental HRQoL** | | |
| --- | --- | --- | --- | --- | --- | --- |
|  | **Mean Difference** | **Lower 95% CI** | **Upper 95% CI** | **Mean Difference** | **Lower 95% CI** | **Upper 95% CI** |
| **Ferritin** |  |  |  |  |  |  |
| <200 | 0.5 (+0%) | -2.4 | 3.3 | 0.1 (+0%) | -2.0 | 2.1 |
| 200-500 | **Ref.** |  |  |  |  |  |
| >500-700 | 0.7 (+0%) | -1.6 | 3.1 | -0.8 (-11%) | -2.5 | 0.8 |
| >700 | -0.6 (-14%) | -3.1 | 2.0 | -1.7 (-6%) | -3.7 | 0.2 |
| **TSAT** |  |  |  |  |  |  |
| <20 | -0.2 (-33%) | -1.9 | 1.5 | -0.4 (+0%) | -1.5 | 0.7 |
| 20-39 | **Ref.** |  |  |  |  |  |
| ≥40 | 0.2 (+0%) | -1.7 | 2.2 | -0.3 (+0%) | -2.4 | 1.9 |

|  | **Fatigue** | | | **Shortness of breath** | | |
| --- | --- | --- | --- | --- | --- | --- |
|  | **Odds Ratio** | **Lower 95% CI** | **Upper 95% CI** | **Odds Ratio** | **Lower 95% CI** | **Upper 95% CI** |
| **Ferritin** |  |  |  |  |  |  |
| <200 | 1.01 (+1%) | 0.60 | 1.69 | 1.05 (+0%) | 0.72 | 1.52 |
| 200-500 | **Ref.** |  |  |  |  |  |
| >500-700 | 1.00 (+0%) | 0.67 | 1.49 | 1.10 (+0%) | 0.82 | 1.47 |
| >700 | 1.00 (-5%) | 0.57 | 1.75 | 1.20 (+0%) | 0.90 | 1.62 |
| **TSAT** |  |  |  |  |  |  |
| <20 | 0.91 (-1%) | 0.66 | 1.25 | 1.04 (+0%) | 0.79 | 1.38 |
| 20-39 | **Ref.** |  |  |  |  |  |
| ≥40 | 0.89 (-1%) | 0.57 | 1.40 | 0.89 (+0%) | 0.61 | 1.31 |

|  | **Muscle cramps** | | | **Restless legs** | | |
| --- | --- | --- | --- | --- | --- | --- |
|  | **Odds Ratio** | **Lower 95% CI** | **Upper 95% CI** | **Odds Ratio** | **Lower 95% CI** | **Upper 95% CI** |
| **Ferritin** |  |  |  |  |  |  |
| <200 | 1.12 (+0%) | 0.86 | 1.47 | 1.01 (+0%) | 0.79 | 1.29 |
| 200-500 | **Ref.** |  |  |  |  |  |
| >500-700 | 1.08 (+1%) | 0.78 | 1.48 | 1.00 (+1%) | 0.64 | 1.55 |
| >700 | 0.90 (+2%) | 0.64 | 1.27 | 1.04 (+0%) | 0.57 | 1.90 |
| **TSAT** |  |  |  |  |  |  |
| <20 | 1.08 (+0%) | 0.86 | 1.36 | 1.00 (+0%) | 0.76 | 1.32 |
| 20-39 | **Ref.** |  |  |  |  |  |
| ≥40 | 0.99 (+1%) | 0.64 | 1.54 | 1.07 (+1%) | 0.73 | 1.55 |

Note: The values in brackets represent the percentage change in the estimates following adjustment for concurrent hemoglobin levels.

**Table S7.** Crude association of iron status with HRQoL and anemia-related symptoms without adjustment for covariates

|  | **Physical HRQoL** | | | **Mental HRQoL** | | |
| --- | --- | --- | --- | --- | --- | --- |
|  | **Mean Difference** | **Lower 95% CI** | **Upper 95% CI** | **Mean Difference** | **Lower 95% CI** | **Upper 95% CI** |
| **Ferritin** |  |  |  |  |  |  |
| <200 | 0.6 | -2.3 | 3.5 | 0.0 | -2.0 | 2.1 |
| 200-500 | **Ref.** |  |  |  |  |  |
| >500-700 | 0.7 | -1.7 | 3.1 | -0.9 | -2.6 | 0.7 |
| >700 | -0.9 | -3.5 | 1.7 | -2.0 | -4.0 | 0.1 |
| **TSAT** |  |  |  |  |  |  |
| <20 | -0.4 | -2.0 | 1.3 | -0.5 | -1.6 | 0.6 |
| 20-39 | **Ref.** |  |  |  |  |  |
| ≥40 | 0.1 | -1.8 | 2.1 | -0.4 | -2.5 | 1.7 |

|  | **Fatigue** | | | **Shortness of breath** | | |
| --- | --- | --- | --- | --- | --- | --- |
|  | **Odds Ratio** | **Lower 95% CI** | **Upper 95% CI** | **Odds Ratio** | **Lower 95% CI** | **Upper 95% CI** |
| **Ferritin** |  |  |  |  |  |  |
| <200 | 1.02 | 0.60 | 1.73 | 1.06 | 0.74 | 1.53 |
| 200-500 | **Ref.** |  |  |  |  |  |
| >500-700 | 1.00 | 0.67 | 1.50 | 1.12 | 0.84 | 1.49 |
| >700 | 1.03 | 0.59 | 1.78 | 1.19 | 0.90 | 1.58 |
| **TSAT** |  |  |  |  |  |  |
| <20 | 0.95 | 0.69 | 1.29 | 1.05 | 0.80 | 1.37 |
| 20-39 | **Ref.** |  |  |  |  |  |
| ≥40 | 0.91 | 0.59 | 1.42 | 0.90 | 0.62 | 1.30 |

|  | **Muscle cramps** | | | **Restless legs** | | |
| --- | --- | --- | --- | --- | --- | --- |
|  | **Odds Ratio** | **Lower 95% CI** | **Upper 95% CI** | **Odds Ratio** | **Lower 95% CI** | **Upper 95% CI** |
| **Ferritin** |  |  |  |  |  |  |
| <200 | 1.12 | 0.85 | 1.48 | 1.02 | 0.79 | 1.30 |
| 200-500 | **Ref.** |  |  |  |  |  |
| >500-700 | 1.09 | 0.79 | 1.50 | 0.99 | 0.64 | 1.52 |
| >700 | 0.89 | 0.63 | 1.24 | 1.03 | 0.57 | 1.88 |
| **TSAT** |  |  |  |  |  |  |
| <20 | 1.07 | 0.85 | 1.35 | 0.99 | 0.75 | 1.30 |
| 20-39 | **Ref.** |  |  |  |  |  |
| ≥40 | 0.99 | 0.64 | 1.53 | 1.07 | 0.74 | 1.53 |

**Table S8.** Baseline characteristics: lost to follow-up vs. not lost to follow-up

|  | **Lost to follow-up**  (n=267) | **Not lost to follow-up**  (n=802) |
| --- | --- | --- |
| **Demographic** |  |  |
| Age, mean (SD) – yr. | 62 (15) | 65 (14) |
| Male sex, no. (%) | 173 (65) | 532 (66) |
| **Clinical** |  |  |
| Hemodialysis, no. (%) | 190 (71) | 613 (78) |
| *Primary kidney disease, no. (%)* |  |  |
| Diabetic kidney disease | 47 (18) | 142 (18) |
| Hypertension | 37 (14) | 139 (17) |
| Renal vascular disease | 20 (8) | 69 (9) |
| Glomerulonephritis | 28 (11) | 100 (13) |
| Pyelonephritis | 17 (6) | 39 (5) |
| Polycystic kidney disease | 15 (6) | 44 (6) |
| Miscellaneous | 47 (18) | 147 (18) |
| Unknown | 56 (21) | 122 (15) |
| *Cardiovascular disease, no. (%)* |  |  |
| Coronary artery disease | 73 (27) | 183 (23) |
| Peripheral artery disease | 70 (26) | 175 (22) |
| Heart failure | 35 (13) | 71 (9) |
| Diabetes mellitus, no. (%) | 82 (31) | 277 (35) |
| Malignancy, no. (%) | 35 (13) | 123 (15) |
| Lung disease, no. (%) | 16 (6) | 73 (9) |
| *Charlson Comorbidity Index, no. (%)* |  |  |
| Low comorbidity score (2 points) | 90 (34) | 238 (30) |
| Intermediate comorbidity score (3-4 points) | 96 (36) | 331 (42) |
| Severe comorbidity score (≥5 points) | 80 (30) | 228 (29) |
| Residual diuresis (>100 mL/day) | 193 (72) | 592 (74) |
| **Laboratory values** |  |  |
| eGFR, median (IQR) – mL/min/1.73m^2^ | 6.2 (8.8) | 6.4 (9.2) |
| Hemoglobin, mean (SD) – g/dL | 9.8 (2.1) | 10.0 (2.1) |
| Ferritin, median (IQR) – ng/mL | 206 (300) | 206 (296) |
| Transferrin saturation, median (IQR) – % | 19 (12) | 18 (14) |
| C-reactive protein, median (IQR) – mg/L | 7.0 (22.6) | 8.0 (23.0) |
| **Antianemia drugs** |  |  |
| IV Iron, no. (%) | 47 (18) | 182 (23) |
| Ferric carboxymaltose, no. (%) | 19 (7) | 85 (11) |
| Iron sucrose, no. (%) | 17 (6) | 45 (6) |
| Iron isomaltoside, no. (%) | 11 (4) | 50 (6) |
| Oral Iron, no. (%) | 22 (8) | 94 (12) |
| ESA, no. (%) | 150 (57) | 442 (55) |
| **Patient-reported outcomes** |  |  |
| *HRQoL* |  |  |
| PCS, median (IQR) – 0 to 100 | 35.0 (13.3) | 34.8 (14.0) |
| MCS, median (IQR) – 0 to 100 | 46.7 (17.0) | 48.3 (15.5) |
| *Anemia-related symptoms* |  |  |
| Fatigue, no. (%) | 185 (85) | 473 (81) |
| Shortness of breath, no. (%) | 77 (35) | 187 (32) |
| Muscle cramps, no. (%) | 122 (56) | 346 (60) |
| Restless legs, no. (%) | 110 (50) | 262 (45) |

^eGFR = estimated glomerular filtration rate (2021 Chronic Kidney Disease Epidemiology Collaboration (CKD-EPI)), IV= intravenous, ESA = erythropoietin stimulating agents, HRQoL = health-related quality of life, PCS = Physical Component Summary, MCS = Mental Component Summary.^

**Table S9.** Sensitivity analysis of the association of iron status with HRQoL and anemia-related symptoms in patients with complete follow-up

|  | **Physical HRQoL** | | | **Mental HRQoL** | | |
| --- | --- | --- | --- | --- | --- | --- |
|  | **Mean Difference** | **Lower 95% CI** | **Upper 95% CI** | **Mean Difference** | **Lower 95% CI** | **Upper 95% CI** |
| **Ferritin** |  |  |  |  |  |  |
| <200 | 0.4 | -2.6 | 3.4 | 0.1 | -2.0 | 2.2 |
| 200-500 | **Ref.** |  |  |  |  |  |
| >500-700 | 0.8 | -1.6 | 3.3 | -1.1 | -2.9 | 0.7 |
| >700 | -0.8 | -3.4 | 1.9 | -1.9 | -4.1 | 0.4 |
| **TSAT** |  |  |  |  |  |  |
| <20 | -0.2 | -1.9 | 1.6 | -0.4 | -1.7 | 0.8 |
| 20-39 | **Ref.** |  |  |  |  |  |
| ≥40 | 0.3 | -1.9 | 2.4 | -0.4 | -3.0 | 2.2 |

|  | **Fatigue** | | | **Shortness of breath** | | |
| --- | --- | --- | --- | --- | --- | --- |
|  | **Odds Ratio** | **Lower 95% CI** | **Upper 95% CI** | **Odds Ratio** | **Lower 95% CI** | **Upper 95% CI** |
| **Ferritin** |  |  |  |  |  |  |
| <200 | 1.02 | 0.60 | 1.74 | 1.05 | 0.71 | 1.56 |
| 200-500 | **Ref.** |  |  |  |  |  |
| >500-700 | 0.99 | 0.64 | 1.53 | 1.09 | 0.80 | 1.48 |
| >700 | 1.06 | 0.57 | 1.96 | 1.18 | 0.88 | 1.60 |
| **TSAT** |  |  |  |  |  |  |
| <20 | 0.91 | 0.67 | 1.25 | 1.03 | 0.78 | 1.36 |
| 20-39 | **Ref.** |  |  |  |  |  |
| ≥40 | 0.86 | 0.52 | 1.43 | 0.91 | 0.59 | 1.41 |

|  | **Muscle cramps** | | | **Restless legs** | | |
| --- | --- | --- | --- | --- | --- | --- |
|  | **Odds Ratio** | **Lower 95% CI** | **Upper 95% CI** | **Odds Ratio** | **Lower 95% CI** | **Upper 95% CI** |
| **Ferritin** |  |  |  |  |  |  |
| <200 | 1.13 | 0.84 | 1.51 | 1.06 | 0.81 | 1.39 |
| 200-500 | **Ref.** |  |  |  |  |  |
| >500-700 | 1.04 | 0.75 | 1.44 | 0.99 | 0.65 | 1.51 |
| >700 | 0.91 | 0.64 | 1.30 | 1.07 | 0.58 | 2.00 |
| **TSAT** |  |  |  |  |  |  |
| <20 | 1.05 | 0.80 | 1.36 | 1.00 | 0.74 | 1.36 |
| 20-39 | **Ref.** |  |  |  |  |  |
| ≥40 | 1.06 | 0.68 | 1.64 | 1.01 | 0.69 | 1.50 |
